# Critical role for p53-serine 15 phosphorylation in stimulating transactivation at p53-responsive promoters

**DOI:** 10.1093/nar/gku501

**Published:** 2014-06-07

**Authors:** Jayne Loughery, Miranda Cox, Linda M. Smith, David W. Meek

**Affiliations:** Division of Cancer Research, Medical Research Institute, The University of Dundee, Ninewells Hospital, James Arrott Drive, Dundee DD1 9SY, United Kingdom

## Abstract

The p53 tumour suppressor is induced by various stress stimuli and coordinates an adaptive gene expression programme leading to growth arrest or cell death. Some stimuli, such as DNA damage, lead to rapid and substantial multisite phosphorylation of p53, nucleated initially through phosphorylation of serine 15. Other stimuli, such as hyper-proliferation, do not stimulate p53-phosphorylation, raising questions regarding the physiological role for phosphorylation. Here, we show that a basal level of Ser15 phosphorylation occurs in both unstimulated cells and cells stimulated pharmacologically to induce p53. p53 in which Ser15 is substituted by alanine (S15A) fails to mediate p53-dependent transcription or growth arrest but can be rescued by substitution with aspartate (S15D: a phospho-mimic). Chromatin immunoprecipitation (ChIP) analyses show that, while wt- and S15A-p53 are detectable on the *CDKN1A* (p21) promoter (as a representative p53-responsive promoter), S15A-p53 does not stimulate histone acetylation (a measure of chromatin relaxation), nor is its recruitment stimulated, in response to a DNA damage or pharmacological stimulus. These data demonstrate that Ser15 phosphorylation is required for p53 function in the physiological context of p53-responsive promoters and suggest a key and possibly universal role even for low levels of this modification in promoting p53-transcription function.

## INTRODUCTION

The p53 tumour suppressor is a short-lived transcription factor that is stabilized and activated in response to a range of cellular stresses including hyper-proliferation and DNA damage (1,2). Induction of p53, regardless of the activating stimulus, is mediated mainly through uncoupling p53 from its key negative regulators, MDM2 and MDM4, leading to the accumulation of stable active p53 (3). Activated p53 coordinates a flexible programme of gene expression that is dependent upon the type and duration of the activating stimulus, the cell type and the growth status of the cell (4,5). This response defines whether the biological outcome of p53 induction will be cell-cycle arrest (transient or permanent) or programmed cell death. However, the molecular mechanisms by which the programme of gene expression varies under different conditions are only partly understood.

At the molecular level, certain stimuli, such as genotoxic stress (DNA damage-inducing agents) and glucose deprivation, promote a series of reversible post-translational modifications (PTMs) of p53 including multisite phosphorylation of the transactivation domain (N-terminus). In addition to contributing towards the induction of p53, these events are thought to regulate p53-mediated transcription at individual promoters, possibly in a selective manner (the ‘barcode’ hypothesis: (4–6)). Serine 15 is the primary target of the DNA damage response on the p53 protein and is phosphorylated by both the ATM and ATR protein kinases (discussed in detail in (7,8)). Similarly, activation of the AMPK protein kinase in response to metabolic stress/glucose deprivation leads to phosphorylation of Ser15 (9).

Biochemically, Ser15 phosphorylation can stimulate association of p53 with important histone/lysine acetyltransferases (HATs), such as p300 and CBP (10–12) Recruitment of these proteins promotes acetylation of multiple lysine residues in the DNA binding and carboxy-terminal domains of p53 and can thus contribute to the stabilization of p53 by blocking ubiquitylation (13,14). However, it has not been established to date whether, in the physiological context of a p53-responsive promoter(s), Ser15 phosphorylation actually leads to corresponding local histone acetylation and relaxation of chromatin as the model predicts, thereby permitting subsequent stimulation of transcription.

Ser15 phosphorylation also triggers a sequential series of additional phosphorylation events in p53 (including phosphorylation of Ser9 -20, -46 and Thr18) that contribute further to p53 induction and activation (14–18). These findings suggest that Ser15 phosphorylation is therefore a major focal point in the activation of p53. Biochemical evidence suggests that these sequential modifications act in the manner of a rheostat by incrementally increasing or decreasing, respectively, association with partners, such as p300 and MDM2, the principal ubiquitin E3 ligase that mediates ubiquitylation and proteasomal degradation of p53 (19–23). Curiously, however, while DNA damage promotes phosphorylation of Ser15 (and indeed other sites in p53), these modifications have not been reported to be stimulated by other p53-activating events, such as the expression of the physiological MDM2 inhibitor, ARF (which is induced by hyper-proliferation) (24,25) or the pharmacological MDM2 inhibitor, Nutlin-3a (24,26). Phosphorylation of p53 has not, therefore, been deemed to be essential for p53 function.

From an *in vivo* perspective, studies with knock-in mice in which Ser18 (the murine orthologue of human Ser15) is substituted by alanine have established that this phosphorylation site contributes to the protection against the late-onset development of a variety of different tumours (27,28). Analysis of cells derived from these mice (murine embryonic fibroblasts [MEFs] and thymocytes) suggest that Ser15 phosphorylation plays a role in selectively regulating p53-dependent transcription in a cell type-dependent manner, at least in murine cells, but the mechanism(s) of this regulation has not been established. In human cancer Ser15 phosphorylation occurs during the earliest stages of tumour development (29) and may, therefore, be an important element in activating p53 tumour suppressor function. However, its contribution to events that occur during cancer development, and which may contribute to tumour suppression, is only poorly understood.

In the present study, we have examined the role of Ser15 modification in a human cell background, in the physiological context of events at p53-responsive genes with particular focus on the well-characterized *CDKN1A* (p21) p53-responsive promoter. We show that Ser15 phosphorylation occurs even in the absence of DNA damage stimuli and that it is required for maximal promoter activity of various responsive promoters. Our data indicate that Ser15 phosphorylation is crucial, not only for DNA damage-induced activation of p53, but also, albeit at a lower level, for p53 activity and biological function in response to the drug Nutlin-3a which acts simply by blocking MDM2-mediated degradation of p53 and is representative of activators that do not normally induce a significant increase in the stoichiometry of Ser15 phosphorylation. Our data also show that Ser15 phosphorylation is required to permit local acetylation of histones and relaxation of chromatin, thereby extending the model whereby this modification promotes interaction with HATs (above), and demonstrate that Ser15 phosphorylation is needed to recruit p53 to the promoter post-stimulus.

## MATERIALS AND METHODS

### Cell culture, mutagenesis and drug treatments

HCT116 (human colon carcinoma, wild-type p53), U2OS (human osteosarcoma-derived, wild-type p53) and H1299 (non-small cell lung cancer derived, p53 negative) cell lines were maintained in Dulbecco's modified Eagle's medium with 10% fatal bovine serum (FBS). Ser15 substitution mutants of human p53 were generated using the Stratagene QuikChange II system as directed by the manufacturer. Clonal (H1299-based) cell lines in which these proteins were expressed in response to isopropyl beta-D-1-thiogalactopyranoside (IPTG) induction were generated using the Stratagene Lacswitch^TM^ system. Clones were selected and maintained in 400 μg/ml hygromycin and 400 μg/ml G418. Unless otherwise stated, clones were treated with 100 μM IPTG for 16 h prior to drug treatments to ensure expression of the relevant p53 protein. IPTG was maintained at 100 μM during subsequent drug treatments.

Drug treatments of cells were carried out at final concentrations and incubation times as indicated in the figure legends. Drugs were made up initially in dimethyl sulfoxide (DMSO) or ethanol and used in independent experiments as a check to ensure that the carrier itself did not stimulate any effects. As controls in several experiments carrier (DMSO or ethanol) was present at a final concentration of 0.1% (v/v).

### Western blotting and antibodies

Membranes were incubated with the monoclonal antibody DO-1 (Moravian Biotechnology), CM1 (a kind gift from Prof. Sir D. Lane) or PAb 1801 (sc-98; Santa Cruz) to assess the presence and levels of p53. Other antibodies used include phospho-p53 (Ser15) (#9284; Cell Signaling Technology), p21 (sc-756; Santa Cruz Biotechnology), H2AX phospho-S139 (ab11174; AbCam), MDM2 (SMP14 and 4B2; Moravian Biotechnology), anti-acetyl Histone H3 (06–599; Upstate) and glyceraldehyde 3-phosphate dehydrogenase (GAPDH) (G8795; Sigma). Western blotting and visualization (chemiluminescence) were carried out using standard procedures.

### Luciferase assays

H1299 cells were seeded at a density of 0.3 × 10^5^ per well onto a 24-well pate. The cells were transfected in triplicate, using Fugene 6 transfection reagent (Roche), with fixed amounts of plasmids encoding luciferase under control of the p53-responsive p21-, BAX- or MDM2-promoters, or the thymidine kinase (TK) promoter as control, together with the SV-*renilla* plasmid for internal standardization. Increasing amounts of plasmids expressing wild type or mutant (S15A, S15D or L22Q/W23S) human p53, or an empty expression vector (pcDNA3), were co-transfected as appropriate. Transfected cells were harvested 24 h post-transfection in Passive Lysis Buffer (Promega) and analysed in a luminometer using the Dual Luciferase Reporter Assay Kit (Promega). Variations in transfection efficiencies were corrected by determining the *Renilla* luciferase activity of the sample.

### Quantitative reverse transcriptase-polymerase chain reaction (RT-PCR)

Total RNA was extracted using the RNeasy kit (Qiagen). cDNA was made with a high capacity RNA-to-cDNA kit (Applied Biosystems). Quantitative polymerase chain reaction analysis (qPCR) was performed with QuantiTect SYBR Green PCR reagent (Qiagen) on Applied Biosciences 7500 Real Time PCR machine. GAPDH was used as an internal control. The primers used are as follows.

*For chromatin immunoprecipitation (ChIP):* ChIP analysis was carried out at three points on the *CDKN1A* gene as defined previously (30). These were at positions -2283, -20 and +8566. The primer used for these analyses were those used by Donner *et al.* (2007) (30) and are given therein.

*For mRNA quantitation* the following primers were used:

CDKN1A (p21): forward - 5′-GGAGACTCTCAGGGTCGAAA-3′

reverse - 5′-GGATTAGGGCTTCCTCTTGG-3′

PUMA: forward - 5′-GACCTCAACGCACAGTACGAG-3′

reverse - 5′-AGGAGTCCCATGATGAGATTGT-3′

BAX: forward - 5′-CCCGAGAGGTCTTTTTCCGAG-3′

reverse - 5′-CCAGCCCATGATGGTTCTGAT-3′

NOXA: forward - 5′-GGTCCCTAATCATGGACTCCC-3′

reverse 5′-CTGTTTGCCAACTTGCTCCAC-3′

GAPDH: forward - 5′-GGCCATGGGAACTTCCCTTA-3′

reverse - 5′-GCCATCCACAGTCTTCTGGGT-3′

### Flow cytometry

Cells were collected and fixed in 80% ethanol for a minimum of 2 h, followed by treatment with 0.2 mg/ml RNase A in phosphate buffered saline. Propidium iodide was added subsequently to a final concentration of 25 μg/ml. The cells were analysed using a Becton Dickenson FACScan system.

### Alamar blue assay

Cell viability was assessed using an Alamar Blue assay as instructed by the manufacturer (Invitrogen).

### ChIP

Following appropriate treatments, cells were cross-linked using 1.5% formaldehyde. All subsequent procedures were performed as described by Metivier *et al.* (31), with Dynabeads (Invitrogen) used instead of protein A-sepharose beads. The PCR primers used were those previously published by Kaeser and Iggo (32).

### Immunoprecipitation

H1299 cells (p53-null) were transiently transfected with plasmids expressing wt-p53, p53-S15A or p53-S15D (encoded in the pCDNA.3 vector) with Lipofectamine 2000 (Invitrogen). Note that 24-h post-transfection the cells were lysed in Igepal lysis buffer, and sonicated. The lysates were then pre-cleared with 50 μl Protein-G agarose beads (Santa Cruz), before being incubated with the appropriate antibody overnight. Immune complexes were precipitated using protein G-sepaharose 4B beads for 1 h at 4°C with gentle rocking. Immune complexes were washed and subsequently analysed by western blotting.

## RESULTS

### Ser15 phosphorylation occurs following p53 induction independently of DNA damage

**Figure 1.**
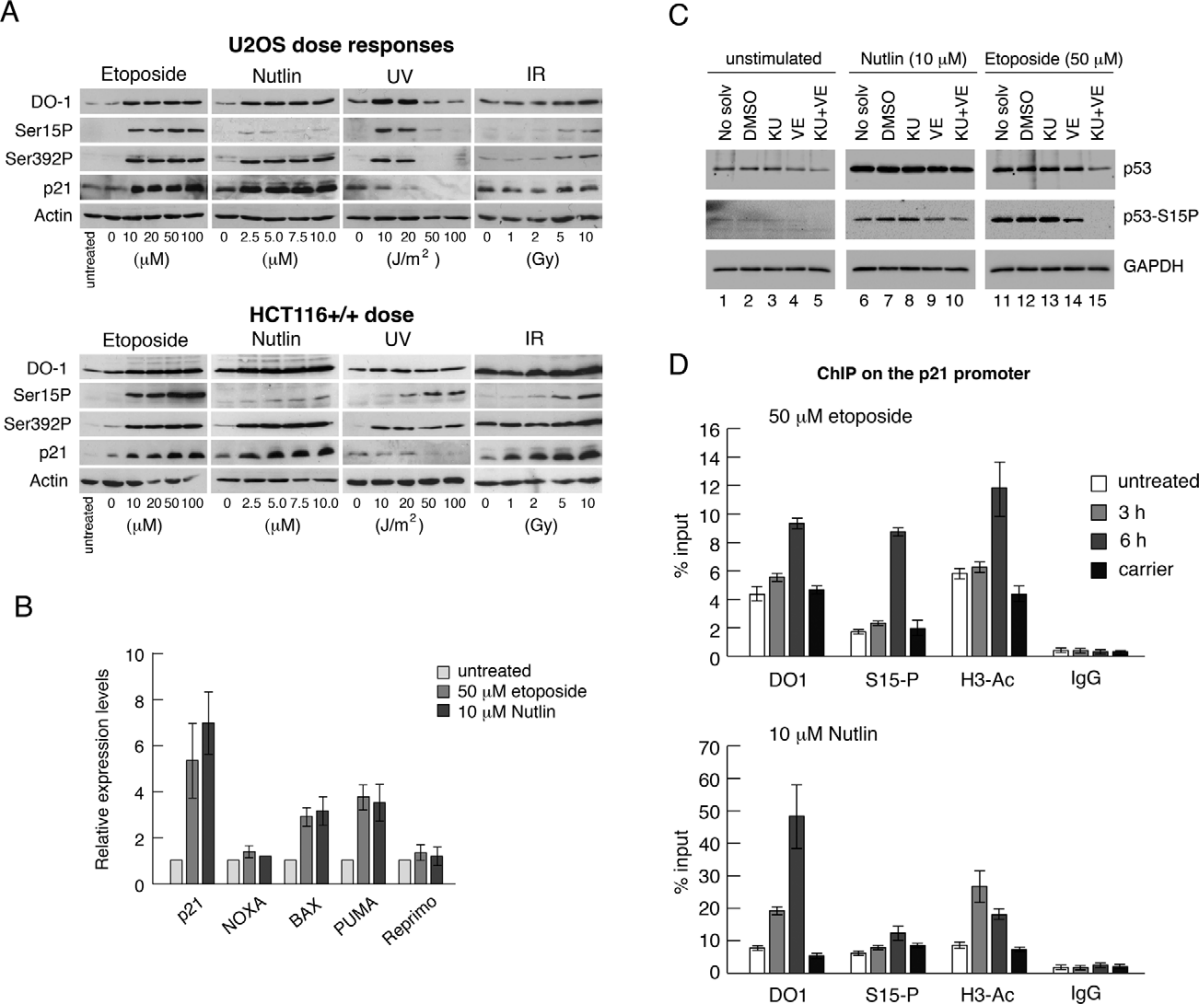
Induction of p53 and p53-phosphorylation by different stimuli. (A) U2OS cells, or HCT116 cells, were stimulated with increasing doses of etoposide, Nutlin-3a, UVC radiation (UV) or ionising radiation (IR). The cells were harvested 6 h after treatment after which various proteins and phospho-proteins were detected by western blotting as indicated. (B) HCT116 cells were treated with 50 μM etoposide or 10 μM Nutlin-3a and harvested after 6 h. The relative levels of expression of various p53-responsive genes were measured by quantitative RT-PCR as indicated. Error bars represent standard deviation over the mean for three separate measurements. (C) HCT116 cells were pre-treated for 1 h with 10 μM KU55933, 10 μM VE821 or with both compounds. The cells were then treated, in the presence of these inhibitors, for a further 6 h with 50 μM etoposide or 10 μM Nutlin-3a, as in panel A, and subsequently harvested. Various proteins and phospho-proteins were detected by western blotting as indicated. (DMSO or ethanol were used as carrier [drug solvent] in independent experiments and were present at a final concentration of 0.1% [v/v]). (D) HCT116 cells were treated for 3 h or 6 h with 50 μM etoposide or 10 μM Nutlin-3a. ChIP analysis was carried out on the p21 promoter (distal site) using the various indicated antibodies.

We recently confirmed that major increases in the levels of p53-Ser15 phosphorylation are induced only by certain stimuli (24). Thus, three DNA damage-inducing treatments that activate p53 (etoposide [a clinically-relevant anti-cancer drug], ultraviolet [UV] and ionising radiation [IR]) induce Ser15 phosphorylation. Nutlin, a pharmacological inhibitor of the p53 negative regulator MDM2, activates p53 without inducing Ser15 phosphorylation (Figure 1A). Curiously, however, p53-dependent gene expression, as measured by the induction of selected p53-downstream genes including the cell-cycle inhibitor p21, appears to be *independent* of increased phosphorylation of p53 ((24) and Figure 1 panels A [protein expression] and B [mRNA levels]), possibly suggesting that Ser15 phosphorylation is dispensable for p53 activity (26). However, there is a low but detectable level of phosphorylated Ser15 in extracts even after induction of p53 by Nutlin-3a ((24); Figure 1A), suggesting that a role for Ser15 phosphorylation cannot be discounted.

Phosphorylation of Ser15 in response to DNA damage is mediated through the ATM and ATR protein kinases (33–35) and can be blocked using highly selective inhibitors of these enzymes (KU55933 and VE821, respectively: Figure 1C). The combined ATR and ATM inhibitors also clearly decreased Ser15 phosphorylation after Nutlin treatment (Figure 1C), suggesting that basal, unstimulated levels of ATM and/or ATR contribute to Ser15 phosphorylation in the absence of DNA damage, at least in cultured cells. Additionally, however, there appears to be a slight amount of remaining phosphorylation in contrast to the situation with etoposide. These data suggest that other protein kinases known to phosphorylate Ser15 (8) may also contribute to its modification in unstimulated and Nutlin-treated cells.

Consistent with the observation that both etoposide and Nutlin treatments activate p53-mediated transcription, p53 recruitment to the distal site on the p21 (*CDKN1A*) promoter (as measured by ChIP) is stimulated by each of these drugs (Figure 1D). Notably, while treatment with etoposide leads to a large increase in the presence of Ser15-phosphorylated p53 at this promoter, there is also a smaller but significant increase in the presence of Ser15-phosphorylated p53 on the p21 promoter following treatment with Nutlin-3a (Figure 1D). The increased signal with the anti-acetylated histone H3 antibody indicates the occurrence of chromatin relaxation and is consistent with increased transcription. These data suggest a potential role for low levels of modification at this site.

### Serine 15 is important for p53-mediated gene expression

**Figure 2.**
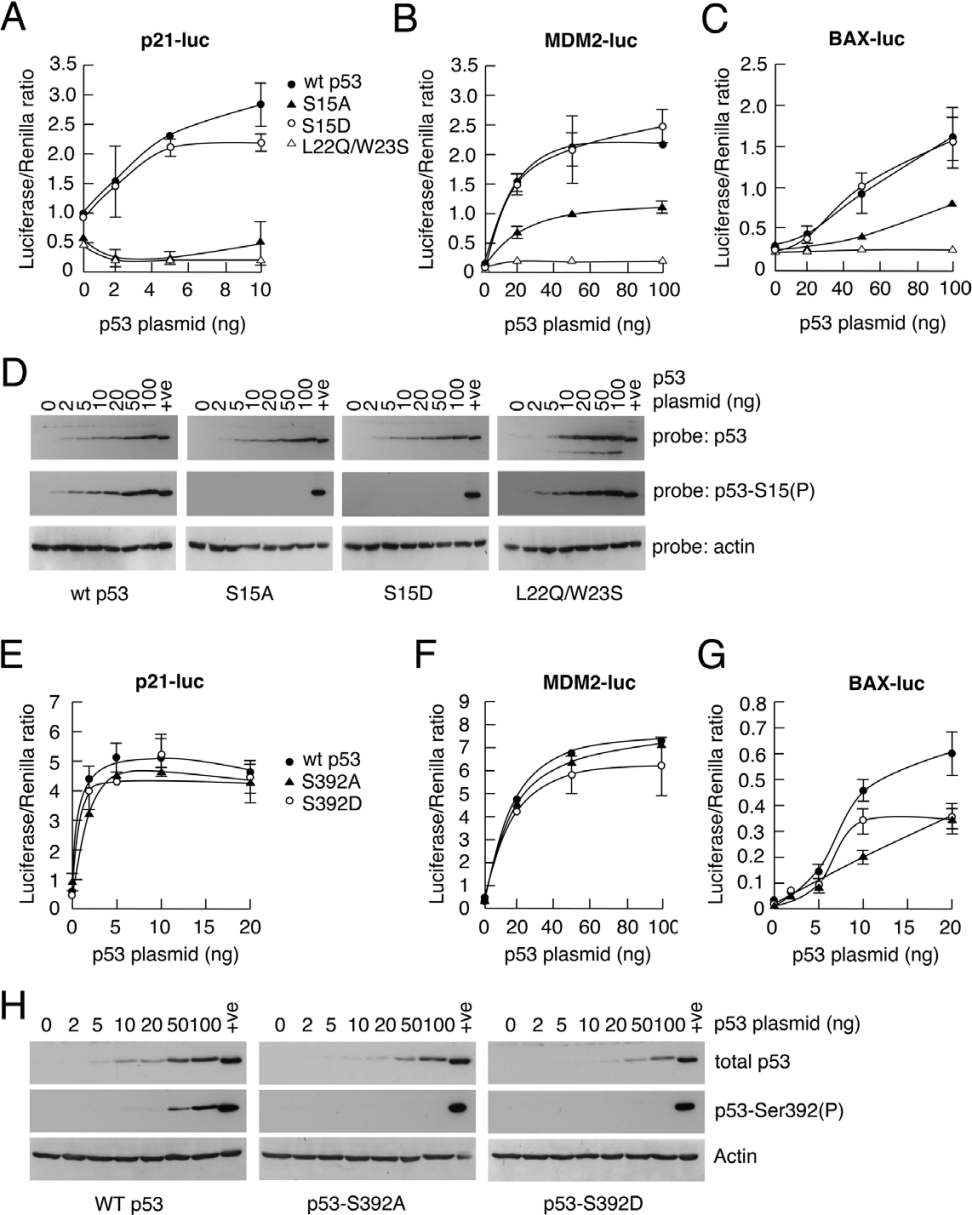
Serine 15 in human p53 is required for efficient p53-mediated transactivation of responsive promoters. Luciferase assays were carried out as described in the Materials and Methods section. In brief, H1299 cells were transfected with plasmids encoding luciferase under control of the p53-responsive p21-, BAX- or MDM2-promoters, together with the SV-*renilla* plasmid for internal standardization. Increasing amounts of plasmids expressing wild type or mutant (S15A, S15D, L22Q/W23S, S392A, S392D) human p53, or an empty expression vector (pcDNA3), were co-transfected as appropriate. The cells were harvested 24 h after transfection. The levels of the expressed p53 proteins were determined for each transfection by western blotting using the antibody DO-1. (In the case of the L22Q/W23S mutant, which is not recognized by DO-1, CM-1 was used for detection.) Since these were consistent from experiment to experiment, typical western blots are shown in panels D and H.

To determine whether Ser15 phosphorylation is important for human p53 function, luciferase assays were carried out in which wild-type p53 and phosphorylation site mutants were compared for their abilities to mediate p53-dependent transactivation. The data (Figure 2) confirmed that wild-type p53 transactivates expression from the p21-, BAX- and MDM2-promoters in a dose-dependent manner (panels A–C, respectively). As control, increasing levels of p53 showed no effect on the non-p53-responsive TK-promoter (data not shown). A mutant p53 protein in which the Ser15 phosphorylation site was substituted by alanine, and which cannot therefore be phosphorylated, showed a significant reduction in its ability to promote expression from the BAX- and MDM2 promoters, consistent with a requirement for Ser15 phosphorylation for full transcriptional activity. Interestingly, the S15A mutant was unable to induce expression from the p21-promoter at any of the levels tested. In all cases a S15D substitution mutant was equally effective at stimulating gene expression as compared with wild-type p53. Given that aspartate or glutamate substitutions can often mimic phosphorylated residues, these data are consistent with the idea that Ser15 phosphorylation is required for effective p53-dependent transactivation. The inclusion of the transcriptionally inactive L22Q/W23S double mutant (36) as a negative control underpinned the requirement for transactivation domain 1 of p53 (TAD1, which contains the Ser15 phosphorylation site) in expression from these promoters. These data strongly suggest that Ser15 phosphorylation is required for p53-dependent gene expression and, given the case of the p21-promoter, suggest that this residue may play a selective and promoter context-dependent role in transactivation.

In contrast to these data, substitution of another major p53 phosphorylation site, Ser392, had no detectable effect on p53 activity under the conditions employed in this study (panels E–G). This observation underpins the specificity and contribution of the Ser15 phosphorylation site. In all cases the levels of expression of the wild-type and mutant p53 proteins were checked to ensure that lack of transactivation in the reporter plasmids could not be attributed to differences in the levels of expression (panels D and H).

### Inducible expression of wild-type and phosphorylation site mutant p53 proteins at near-physiological levels highlights a crucial role for Ser15 phosphorylation in mediating growth arrest

**Figure 3.**
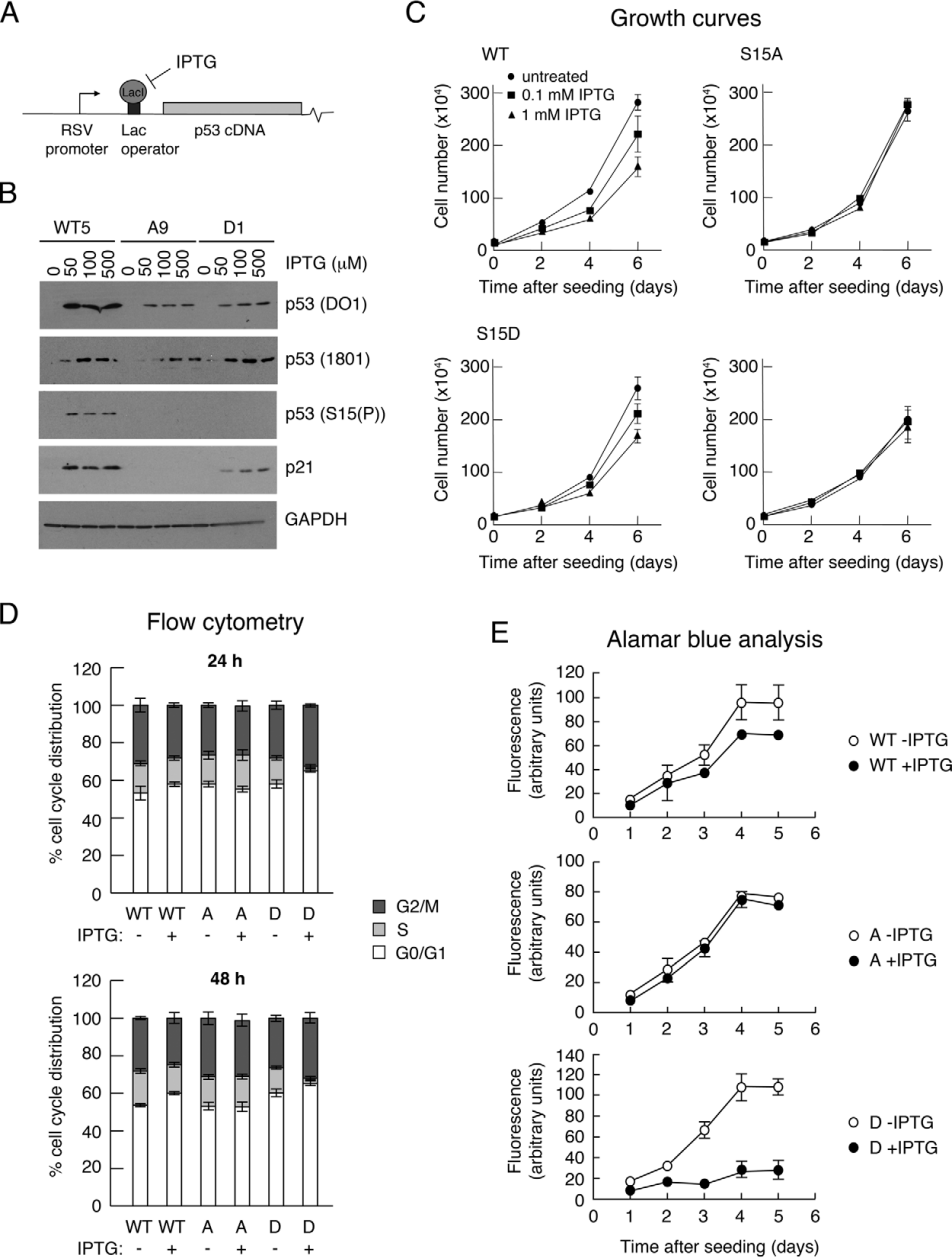
Clones of H1299 cells expressing wild-type or phosphorylation site-mutant p53 proteins. (A) Representation of p53 cDNA under inducible control from the RSV promoter in the LacSwitch II system. (B) Western blot analysis of typical p53-expressing clones following induction by increasing concentrations of the inducer, IPTG. (C) Growth curves of typical p53-expressing clones in the absence and presence of increasing doses of the inducer, IPTG. The cells were seeded at 5 × 10^4^ cells per dish and treated with 0, 0.1 or 1 mM IPTG. The cells were harvested and counted at 2-day intervals for up to 6 days. (D) Cell-cycle distribution of clones in the absence and presence for 24 h (upper panel) and 48 h (lower panel) of 1 mM IPTG. (E) Alamar Blue growth curves of typical clones in the absence and presence of 1 mM IPTG.

While the data from the luciferase assays give a strong indication that Ser15 phosphorylation has profound effects on downstream p53 transcription, they rely on ectopic expression of p53 and the use of reporter plasmids as targets. It is, therefore, important to explore the influence of Ser15 phosphorylation on endogenous p53-responsive genes. However, given that the levels of endogenous p53 proteins play a critical role in p53-mediated gene expression and biological outcome, it is necessary to be able to express the phosphorylation substitution mutants at physiological levels in all cells of a given population. To address this problem, clones of H1299 cells ectopically expressing wild-type or mutant p53 using the IPTG-inducible LacSwitch II system were generated and screened, as described in the Materials and Methods section (Figure 3A). (We have used this system previously to investigate the contribution of p53 phosphorylation to regulation of BCL-3 expression (37). H1299 cells are ideal for this purpose because they lack endogenous p53 expression.) The advantage of this system is that it allows low but controllable levels of expression that can be adjusted to match the levels of expression of endogenous p53 in other established cultured cell lines.

Three clones expressing wt human p53, three expressing the S15A mutant and three expressing the S15D mutant were adopted for further analysis. For presentation purposes, the data from only one of each type (WT5, A9 and D1) are shown in the figures, unless stated otherwise, but are representative of similar observations made using the other clones. All clones showed p53 expression that is inducible by IPTG (Figure 3B) and detectable by two independent p53-specific monoclonal antibodies with different epitopes (DO1 and PAb1801). Notably, Ser15 phosphorylation was detectable in the absence of any stress stimulus and was observed only with wt p53 but not with the S15A or S15D proteins. Consistent with the luciferase data, both the wt and S15D proteins stimulated the levels of endogenous p21. However, p21 was barely detectable in the S15A clone.

To determine whether the expression of the p53 proteins had any biological effects on cell status, growth curves were carried out. These data (Figure 3C) show that IPTG stimulation (and consequent p53 expression) led to a reduced growth rate in the case of the wt p53- and S15D-expressing clones but not the S15A or, importantly, the parental H1299 cells, both of which were unaffected by IPTG treatment. Consistent with these findings, cell-cycle distribution analyses, 24 and 48 h after IPTG treatment (Figure 3D), showed an increase in the proportion of G1 cells and corresponding decrease in the S phase population in the wt p53-expressing clones but no change in the cells expressing the S15A mutant. The cells expressing the S15D mutant, which, in the absence if IPTG, had an increased G1 population as compared with the wt p53-expressing cells, showed a remarkable increase the G1 and G2/M populations that was accompanied by a significant reduction in S phase cells. Notably, there was little increase in the sub-G1 cell population suggesting that, in the H1299 cell system, the induction of p53 led principally to growth arrest and not cell death. Alamar Blue analysis confirmed that, following IPTG treatment, the wt p53 and, particularly, the S15D mutant, significantly down-regulated cell proliferation, whereas the S15A mutant showed no measurable effect. These clonal lines can, therefore, mimic the induction of endogenous p53 and underscore the importance of Ser15 phosphorylation in mediating growth arrest.

### S15A mutant proteins fail to support p53-dependent transcription in response to Etoposide or Nutlin

**Figure 4.**
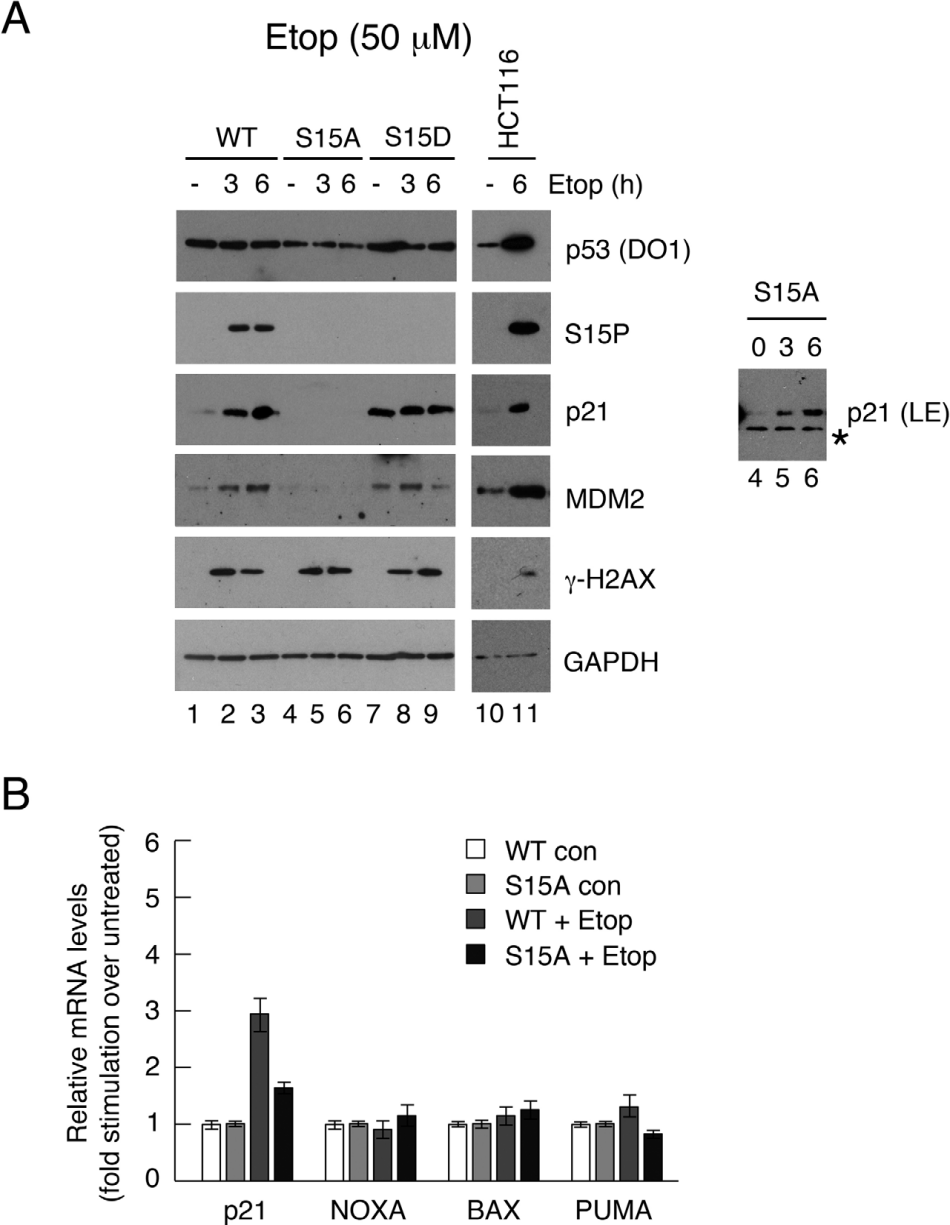
Effect of etoposide on p53 downstream gene expression in H1299 cell clones expressing wild-type or phosphorylation site mutants of p53. (A) Clones of H1299 cells were pre-treated with 100 μM IPTG to permit expression of the wild type, S15A or S15D p53 proteins. The cells were then treated for up to 6 h with 50 μM etoposide and subsequently harvested. Various proteins and phospho-proteins were detected by western blotting as indicated. HCT116 cells were treated in a similar manner to compare the p53 levels and responses with an endogenous p53 response. (B) The levels of expression of various p53-responsive genes before and after the etoposide treatment were measured by quantitative RT-PCR in the wt and S15A clones. The levels achieved following drug stimulation are expressed relative to the uninduced levels in the same clonal line.

To determine whether substitution of Ser15 phosphorylation had any influence on the ability of p53 to stimulate endogenous gene expression, clonal lines were treated with IPTG to induce expression of wt, S15A or S15D p53 and were subsequently further treated with etoposide for up to 6 h. The data (Figure 4A) indicate that the short etoposide treatment did not increase the p53 levels in any of the clones over and above those obtained with the IPTG. The wild type clone showed a dose-dependent stimulation of p21 protein levels. In the S15D clone, p21 levels are high, even in the absence of etoposide, and are not significantly increased by etoposide treatment, consistent with the idea aspartate is mimicking constitutive phosphorylation at this site. In contrast, p21 expression was barely detectable in the S15A clone. Interestingly, however, prolonged exposure of a p21 western blot revealed that there was still a degree of p21 inducibility following etoposide treatment, albeit starting from a very low basal level (panel A inset). Notably, the levels of p53 expression, and the downstream response, achieved using the LacSwitchII system were broadly similar to the endogenous p53 response seen in HCT116 cells. These data underpin the value of using this inducible system to study the human p53 phosphorylation site mutants.

To confirm that the lack of induction of p21 protein in the S15A mutant reflected a reduced stimulation of mRNA levels, comparative analysis of selected p53-dependent gene expression in the wt and S15A clones was carried out using quantitative RT-PCR. The data confirmed that p21 expression failed to show any response to induction of the S15A mutant (Figure 4B). Similarly, the small induction of PUMA seen in this cell background in response to etoposide was absent in the S15A-expressing cells. Notably, BAX and NOXA were not significantly induced in this context.

**Figure 5.**
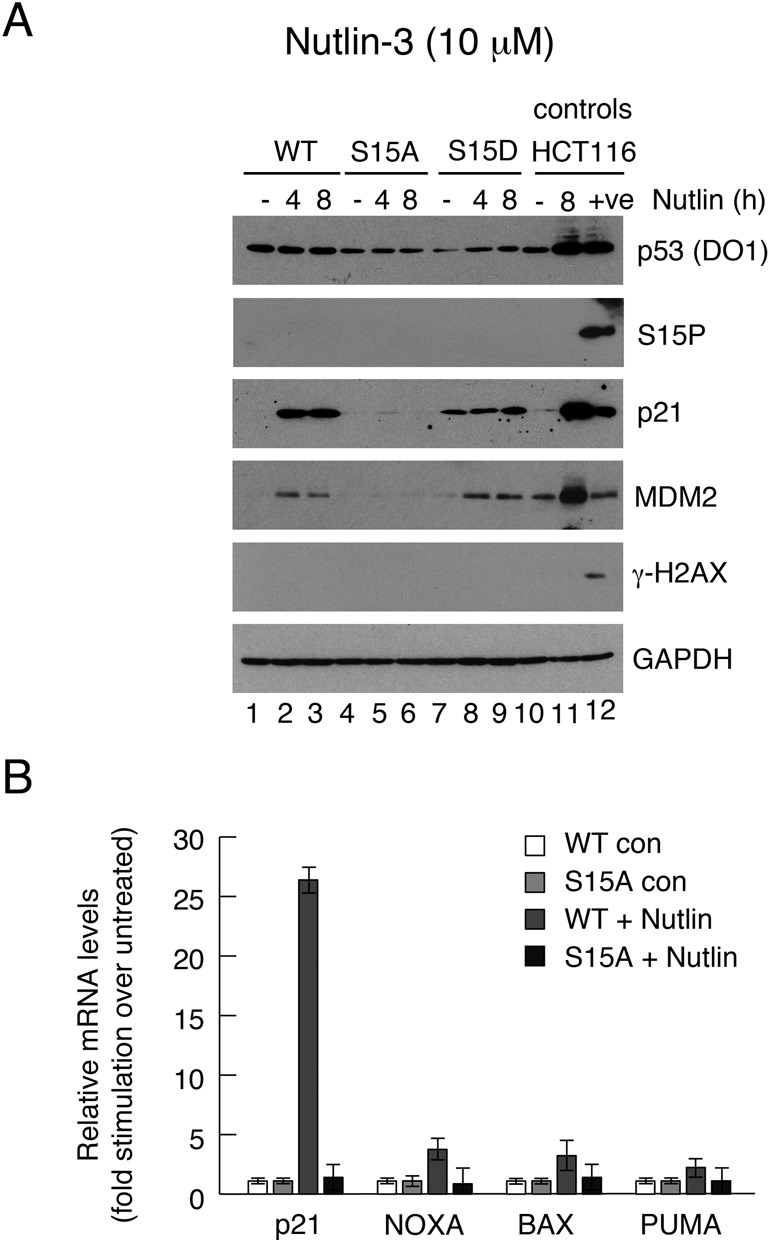
Effect of Nutlin-3a on p53 downstream gene expression in H1299 cell clones expressing wild-type or phosphorylation site mutants of p53. Clones of H1299 cells were pre-treated with 100 μM IPTG to permit expression of the wild type, S15A or S15D p53 proteins. The cells were then treated for up to 8 h with 10 μM Nutlin-3a and subsequently harvested. Various proteins and phospho-proteins were detected by western blotting as indicated. HCT116 cells were treated in a similar manner to compare the p53 levels and responses with an endogenous p53 response. The ‘+ve’ lane is an additional control in which the HCT116 cells were treated with 50 μM etoposide (as in Figure 5). (B) The levels of expression of various p53-responsive genes before and 6 h after the Nutlin treatment were measured by quantitative RT-PCR in the wt and S15A clones. The levels achieved following drug stimulation are expressed relative to the uninduced levels in the same clonal line.

Similar experiments were conducted in which p53 was induced using Nutlin-3a (Figure 5), which works by inhibiting MDM2 and does not stimulate a major increase in p53 phosphorylation (38). Again, while p21 and MDM2 were induced in the wt p53 and S15D clones, the levels of these proteins were very low in the S15A clone (Figure 5A). These data are consistent with a requirement for Ser15 phosphorylation for p53 activity, even when the inducing agent is not one that normally stimulates high levels of phosphorylation. Comparative analysis of selected p53-dependent gene expression in the wt and S15A clones by quantitative RT-PCR showed that, in response to Nutlin, all of the genes (p21, BAX, NOXA and PUMA) were induced by wt p53 but, strikingly, were unaffected in the cells expressing the S15A mutant p53 (Figure 5B). The significant differences in the magnitude of induction of these genes in response to maximal levels of Nutlin as compared with maximal levels of etoposide (Figure 4) may simply reflect characteristics of a particular dose of inducer. Alternatively, it is possible that these activators may affect p53-mediated transcription through their established influence on other cross-talking pathways (see Discussion).

### Ser15 is required for efficient recruitment of p53 to chromatin

**Figure 6.**
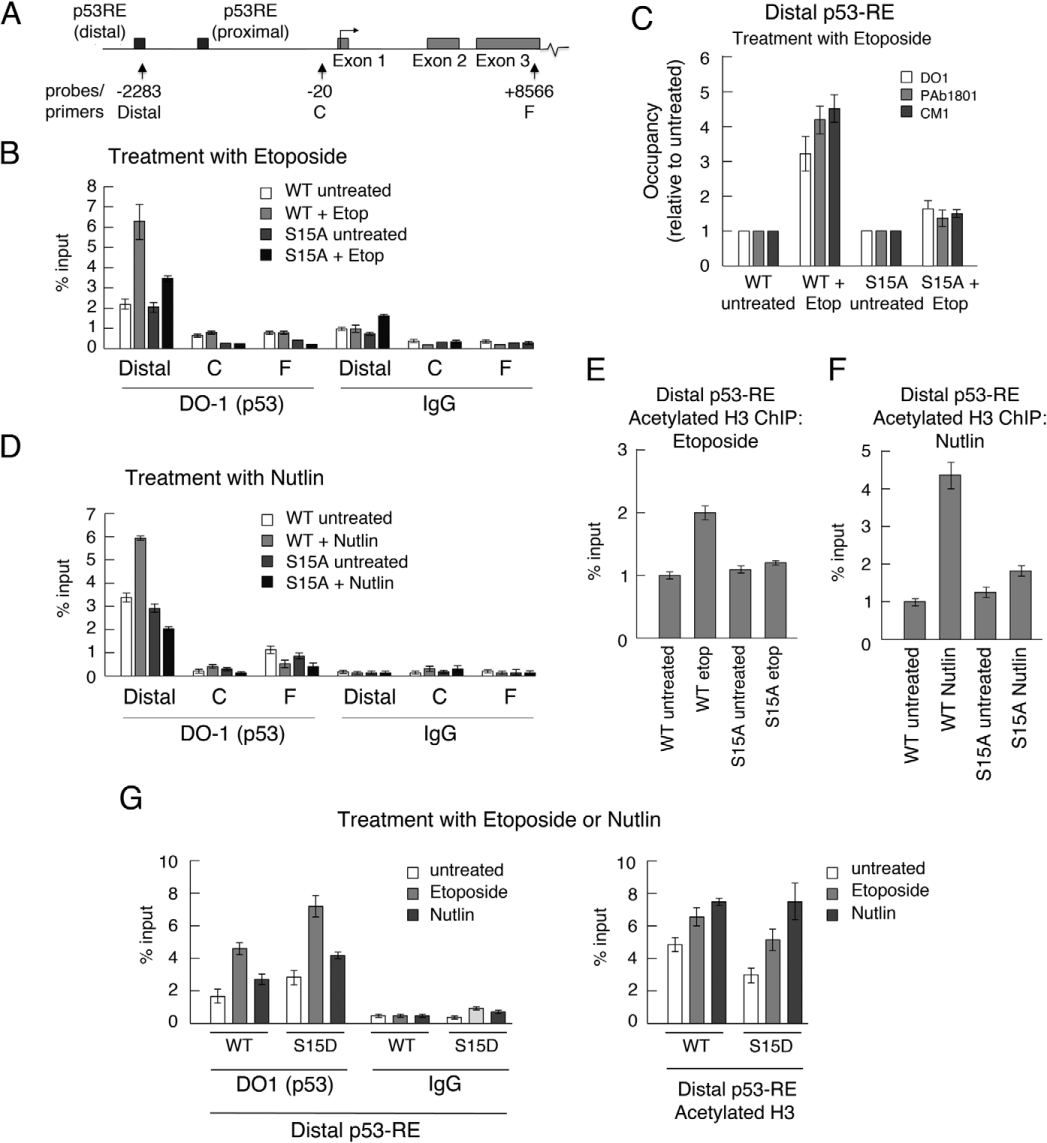
Serine 15 substitution inhibits association of p53 with the p21 promoter. (A) Schematic representing the p21 gene indicating the positions of the probes and primers used in this study. (B) The levels of p53 recruited to the distal p53-responsive element (Distal), the core promoter (C) and the 3′ end (F) of the *p21* gene in H1299 cells expressing WT- and S15A-p53, before and 6 h after treatment with etoposide, were measured by quantitative ChIP using the anti-p53 antibody DO1. (Cells had been pre-treated with 100 μM IPTG prior to etoposide treatment to permit expression of the p53 proteins.) Data points were obtained in triplicate and mean values with associated errors are shown. As control, the DO-1 antibody was substituted with a non-specific IgG. (C) Comparative ChIP analysis of the Distal response element was carried out as in panel B using three independent anti-p53 antibodies (DO-1, PAb1801 and CM-1). With each antibody the increased occupancy following stimulation is presented relative to the untreated samples. (D) Comparative ChIP analysis of the Distal response element was carried out as in panel B except that Nutlin was used as the stimulus in place of etoposide. (E and F) The levels of acetylated histone H3 were determined by ChIP using an anti-acetylated histone H3 antibody (Ac-H3). The cells were stimulated with etoposide (E) or Nutlin (F). (G) The levels of p53 (left-hand panel) and acetylated H3 (right-hand panel) at the Distal p53-RE of the *p21* gene in H1299 cells expressing WT- or S15D-p53 before and 6 h after treatment with etoposide, were measured by quantitative ChIP as above.

It is well established that Ser15 phosphorylation stimulates the interaction of p53 with key transcriptional proteins (8,39) but how modification of this residue regulates events in the physiological context of chromatin has not been established. To explore the role of Ser15 modification in switching on p53 function, comparative ChIP experiments were carried out using the wt p53- and S15A-expressing clones and focusing on the p21 promoter as a model promoter. Examination of recruitment of p53 to three sites was carried out: the distal p53-responsive element (at −2283 bp from the transcriptional start site: Distal), and, as controls, at the core promoter (−20 bp: C) and the 3′ end of the gene (+8566: F) (Figure 6A).

The data show that, in response to etoposide treatment, p53 was recruited to the distal element of the p21 promoter but was not detectable at either the core promoter or the 3′ end of the gene (Figure 6B). In the unstimulated cells, the level of occupancy of wt p53 and the S15A mutant were similar; (p53 has previously been shown to occupy this site prior to any activating stimulus (40)). Interestingly, however, following stimulation with etoposide, while the recruitment of the wild-type proteins was stimulated by several fold, recruitment of S15A-p53 was significantly weaker, suggesting that Ser15 phosphorylation is required for association of p53 with this promoter post-induction. The control analyses confirmed that p53 was not recruited to the core promoter or coding region of the gene.

When different p53 antibodies recognising different p53 epitopes were used in the ChIP experiment to immunoprecipitate the p53, similar data were obtained (panel C), underpinning that the result was not antibody-specific nor was it the consequence of masking and/or modification of an epitope. Importantly, the acetylation of histone H3, a marker for histone acetylase (e.g. p300/CBP) recruitment, chromatin relaxation and transcription activation, was stimulated within the vicinity of the distal responsive element only in the case of the wt p53 (panel E). These data suggest, mechanistically, that the lack of Ser15 phosphorylation led to a failure to engage this important step in transcriptional activation. The lack of histone acetylation observed with the S15A mutant is consistent with the biochemical model that Ser15 phosphorylation plays a key role in mediating association with HATs (10–12,19–23). When Nutlin was used as the stimulus in place of etoposide, very similar results were obtained (Figure 6D and F).

The recruitment of the S15D mutant to the p21 promoter was also examined (Figure 6G). The data indicate a slightly stronger occupancy than wild-type p53, consistent with a requirement for phosphorylation of Ser15. The data also show that, as with wild-type p53, the recruitment is further increased following etoposide or nutlin treatment. These findings suggest that additional factors (such as increases in p53 levels or subsequent additional PTMs) are likely to contribute to the stimulus-dependent increase in recruitment. Taken together, these observations demonstrate that Ser15 phosphorylation is required for p53-mediated p21 expression even by stimuli that do not give rise to major increases in phosphorylation.

### Serine 15 substitution does not alter the conformational status of p53

**Figure 7.**
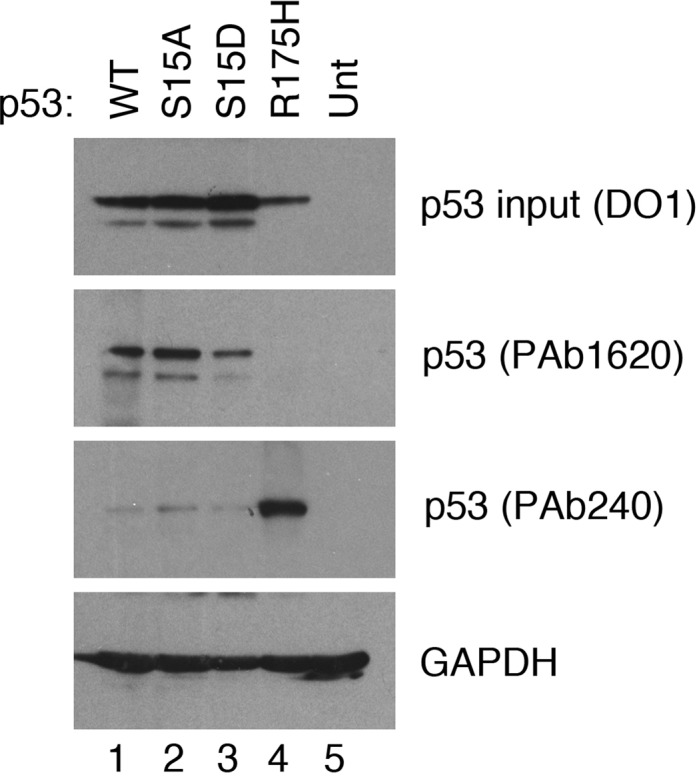
Serine 15 substitution does not alter the conformational status of p53. Plasmids expressing WT p53, p53-S15A, p53-S15D or the conformational mutant p53-R175H (as control) were transfected into H1299 cells. 8 h after transfection, lysates were prepared and the p53 proteins immunoprecipitated using the conformation-specific antibodies, PAb1620 (WT conformation-specific) or PAb240 (mutant conformation-specific). The immunoprecipitated proteins were detected by western blotting using the DO-1 antibody.

The site-specific binding of p53 to DNA relies upon the core (DNA binding) domain of p53 adopting an appropriate conformation; (the so-called ‘wild type’ conformation). p53 conformational status is flexible in cells, is temperature-dependent and can be influenced by several factors (41). Moreover, many mutations that occur within the core domain in human cancers shift the equilibrium away from a DNA binding conformation to the so-called ‘mutant’ conformation. Given the importance of conformational status for DNA binding, the conformations of wild-type p53, together with the S15A and S15D mutant proteins was assessed using the anti-p53 conformation-specific antibodies PAb1620 (which recognizes p53 only in its ‘wild type’ [site-specific DNA binding] conformation) and PAb240 (which recognizes an epitope in p53 that is masked when the protein adopts the wild type conformation). Analysis of cell extracts (Figure 7) revealed that PAb1620 was able to immunoprecipitate the S15A and S15D mutant p53 proteins as effectively as wild-type p53. PAb240 recognizes only a small proportion of each of the three proteins. An R175H mutant p53 protein was immunoprecipitated only by the PAb240 antibody, thereby confirming the specificity of these conformation-specific antibodies. These data indicate that Ser15 modification is highly unlikely to affect association of p53 with chromatin by regulating changes in the conformation of the p53 core domain.

## DISCUSSION

### The significance of Ser15 phosphorylation in p53

Ser15 is a major target for phosphorylation in the p53 protein. In the present study, we show that the contribution of this pivotal modification is much wider than had previously been anticipated. The significance of our findings are essentially 3-fold. (i) We show for the first time that a model for the regulatory role of this phosphorylation site, previously developed on the basis of biochemical analyses, operates within the physiological context of p53 transactivation function at p53-responsive promoters, i.e. at the major sites of p53 biological activity. (ii) We also find that Ser15 phosphorylation is important not only for the recruitment of histone acetyl-transferases to promoters, leading to chromatin relaxation, but also plays an unexpected role in the recruitment of p53 itself to chromatin. (iii) Crucially, our data overturn the assumption that the regulatory contribution of Ser15 phosphorylation is restricted to only certain (i.e. DNA damage and glucose depletion) of the many stimuli that activate p53, and reveal that this important PTM plays a fundamental role in regulating p53 function irrespective of the intitiating stimulus.

### Ser15 phosphorylation is required for p53 function in the physiological context of p53-responsive promoters

**Figure 8.**
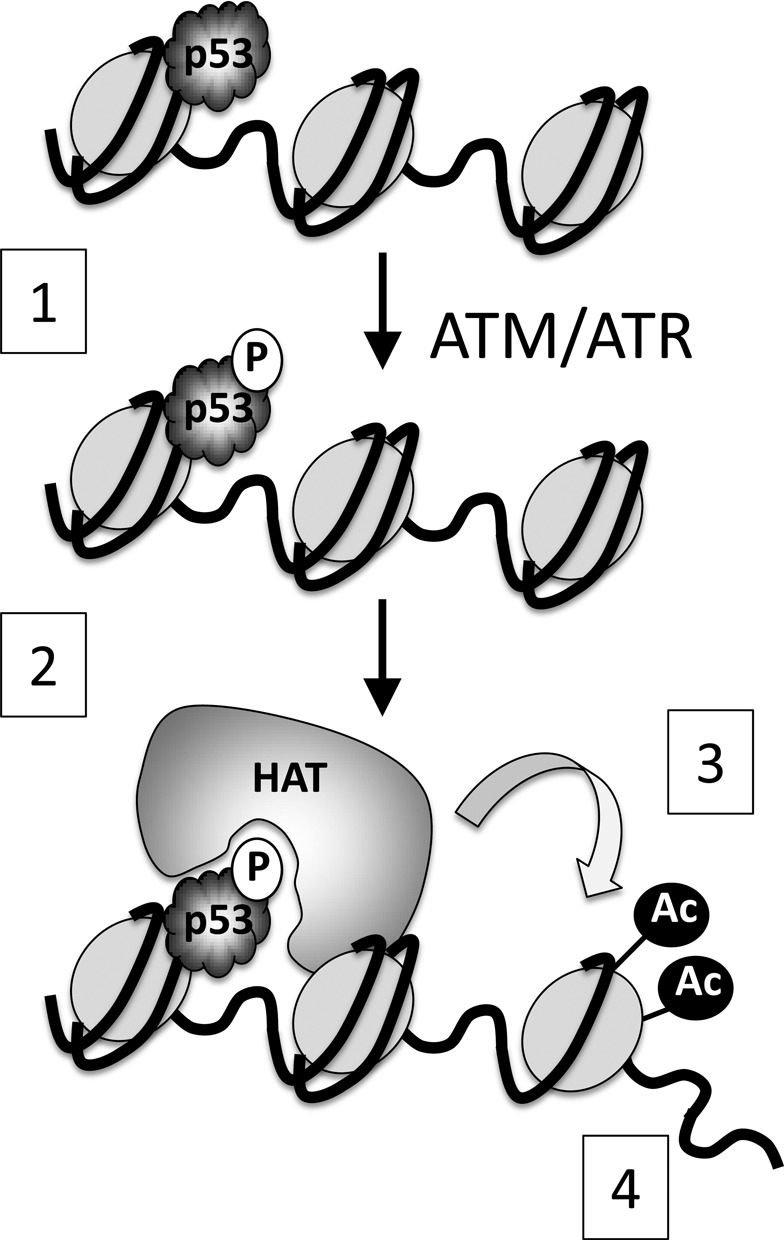
Schematic describing a model for the role of Ser15 phosphorylation in p53-mediated transactivation. (1) p53 undergoes phosphorylation on Ser15. (2) This permits recruitment of histone acetyl-transferases (e.g. p300) (2) leading to acetylation of histones in the neighbouring vicinity (3) and subsequent relaxation of the chromatin (4). The inability to phosphorylate p53 at residue 15 (e.g. in the S15A mutant) impedes HAT recruitment and subsequent histone acetylation.

Ser15 phosphorylation is robustly induced by some stress stimuli (such as DNA damage) but not by others (7,8). Here, we show that both basal (uninduced) p53, and indeed p53 that is induced simply through inhibition of MDM2, show detectable levels of Ser15 phosphorylation, at least in cultured cells. To address a possible role for this modification in a physiological context we generated cell lines that express levels of wild type or phosphorylation site-substitution p53 proteins, in response to IPTG treatment, that match the levels of p53 seen in commonly used p53-responsive human cell lines. (We have used similar lines previously to study the regulation of cyclin D1 expression by p53 (37).) The data generated from the analysis of these lines have established several important principles: (i) Ser15 phosphorylation is required for full p53 transcriptional activity and for its biological response, at least in terms of cell-cycle arrest. The use of a phospho-mimic (the S15D substitution mutant), which rescues the transcriptional activity and cell-cycle arrest functions absent from the S15A mutant, strongly suggests that the biological requirement for Ser15 lies its capacity to act as a major phosphorylation site. (ii) A model based on these findings, describing the role of Ser15 phosphorylation within the physiological context of chromatin, is entirely consistent with previously published biochemical data showing that the modification promotes tight association with histone acetyl-transferases, such as p300/CBP (10–12,19–23) (Figure 8 and see below). (iii) While some stress stimuli, such as DNA damage, promote significant increases in the stoichiometry of Ser15 phosphorylation, there does indeed appear to be a universal requirement for phosphorylation of this residue in maintaining full p53 transcriptional and biological activity, even when p53 is induced by stimuli that do not normally stimulate its phosphorylation. This would imply that phosphorylation of Ser15 mediates possibly two separable functions of p53: one of these underpins basic transactivation function (part of which is HAT recruitment (10–12)) and is likely to be common to all modes of activating p53. At the same time, however, the DNA damage and glucose deprivation pathways stimulate large increases in the level of Ser15 phosphorylation: such increases may reflect true increases in stoichiometry of phosphorylation (e.g. they could be a measure of how many subunits within the p53 tetramer are phosphorylated). Alternatively, given that the level of phosphorylation measured in cell extracts reflects an ‘average’ of all of the p53 molecules in the cell, the increased phosphorylation could be a representation of the length of time p53 molecules exist in the phosphorylated state. While the present study does not experimentally address any possible second role for Ser15 phosphorylation, the striking difference in the levels and/or duration of Ser15 phosphorylation induced by etoposide as compared with Nutlin suggests that there is a specific need for these higher levels, such as mediating in full the events set in motion by the genotoxic stimulus. For example, it is well established that different stress stimuli that impact p53 promote the assembly of transcriptional complexes that differ in their constituent components (40). It is, therefore, possible that increased or prolonged phosphorylation of Ser15 may be necessary to permit the required interactions promoted by some stress stimuli but not by others. Moreover, in addition to acetylating various C-terminal lysine residues in p53, and in modifying neighbouring histone proteins, CBP/p300-mediated acetylation of K164 in the DNA binding domain of p53 is important for the expression of genes involved in growth arrest (42). Increased phosphorylation could have an impact on the outcome of recruiting CBP/p300, such as the relative extent to which these proteins acetylate specific sites within p53 or indeed the neighbouring histones. These are issues which future analysis of the role of Ser15 phosphorylation should address.

Curiously, we have also found consistently in this study that the maximum level of p53-dependent gene expression achieved following Nutlin treatment is significantly greater in magnitude than that seen maximally with etoposide. The most plausible explanation for this observation lies in the ability of these p53 activators to affect other pathways. For example, the NFκB pathway, which is also activated by DNA damage, generally acts through several mechanisms to down-regulate p53 transcriptional activity. In response to DNA-damaging agents, such as etoposide, IKKα phosphorylates CBP on two serines which prevents CBP from interacting with p53 (43). CBP and p300, which are thought to be limiting components for transcriptional activation, are therefore likely to be rate-limiting for p53-mediated transcription following DNA damage, especially in HCT116 cells which are hemizygous for mutant p300 (44). Nutlin, on the other hand, downregulates TNF-α-induced activation of NFκB, at least in some cells (45), and may therefore prevent sequestration of CBP or other factors from p53.

### A model for the role of Ser15 phosphorylation in regulating p53 transcription function

From a mechanistic perspective, the data define a model for the requirement for Ser15 phosphorylation in the physiological context of mediating changes in chromatin that promote transactivation. This model states that the recruitment of p53 to promoters under non-stimulation conditions (at least the *CDKN1A* [p21] promoter, which is the focus of the present study) occurs essentially independently of Ser15 phosphorylation. In support of this conclusion, we find that (i) the ChIP data show roughly equal levels of wt or S15A p53 on the p21 promoter prior to a DNA damage or pharmacological stimulus (Figure 6 panels B and D); and (ii) the so-called ‘wild type’ (site-specific DNA binding) conformation of p53 is unaltered by substitution of Ser15 (Figure 7). Upon stimulation, wild-type p53 mediates transactivation of p21 (Figures 4 and 5): this requires recruitment and/or activation of HAT function as shown by the increased histone acetylation in the immediate vicinity of the distal p53-binding site (ChIP data: Figure 6 panels E–G). Notably, the S15A mutant p53, while located at the p53 binding site, is unable to mediate histone acetlyation and is unable to promote transactivation, even in unstimulated conditions (Figures 4 and 5). These observations are entirely consistent with the published biochemical data (10–12,19–23) showing that Ser15 phosphorylation is required for optimal interaction with the HATs CBP and p300 (the major HAT involved in p53-dependent relaxation of chromatin at the p21 promoter (46)).

It is not clear why the increased recruitment to the p21 promoter observed with the wild-type p53 protein is absent with the S15A mutant. One possibility is that the rate of accumulation of S15A p53 on the promoter is much slower that that of the wild type protein. The on-rates for p53 site-specific DNA binding have been reported to be exceedingly fast (47,48). This, taken together with the known ‘wild type’ conformation of the S15A p53 (Figure 7), suggests that direct DNA binding is highly unlikely to be affected by the S15A substitution. Given, however, that p53 binds significantly more tightly to chromatin than to naked DNA (46), the phosphorylation may speed up recruitment by helping anchor p53 to nearby nucleosomes and/or components of the transcription machinery. It is also possible that the assembly of complexes with transcriptional proteins may reduce the off rate for p53 binding: in support of such an idea it was previously established that the binding of antibodies to epitopes in the N-terminus of p53 can significantly reduce p53 dissociation from the DNA (48). Alternatively, the increased wt- but not S15A-p53 association with the promoter could be explained if the promoter exists in two states within a cell population, one of which is open and permissive for binding both p53 species (Ser15-phosphorylated and unphosphorylated), and a second fraction that requires remodelling before p53 can bind effectively. Wt p53 was recently shown to have properties of a pioneer transcription factor and is capable of binding to responsive elements on the surface of nucleosomes (49). Thus, if Ser15 were critical for this pioneering activity, then only wt p53 would show increased binding and transactivation after treatment.

### Potential relevance of Ser15 phosphorylation to wider p53-mediated events

In addition to its classical role in responding to cellular stresses, such as DNA damage, p53 is now known to make important contributions to a range of homeostatic processes including maternal reproduction, metabolism and, importantly, the biology of stem cells where it can impact on processes, such as self-renewal, differentiation and plasticity (50). A key feature of the ability of p53 to regulate these aspects of stem cell function is its ability to down-regulate the expression of critical genes, such as Nanog and Oct4, which drive embryonic stem cell self-renewal and dedifferentiation. The crucial role of stem cells, both in embryogenesis and in adult tissue and organ renewal, necessitates high fidelity in maintaining genomic stability and in responding to the occurrence of genetic lesions. It will be interesting, therefore, to determine whether the model defining the function of Ser15 phosphorylation (Figure 8) also impacts on the regulation of genes involved in these other processes by governing the recruitment of p53 and defining its interaction with relevant partner proteins. This would be of particular interest given the involvement of other DNA damage-inducible phosphorylation sites in p53 in stem cell biology. For example, Ser315 phosphorylation plays a role in controlling Nanog expression (51). Moreover, mutant mice harbouring aspartate substitutions in two key DNA damage-inducible phosphorylation sites, Thr21 and Ser23 (equivalent to human Thr18 and Ser20), show depletion of multiple stem cell populations, mainly through p53-mediated expression of PUMA (52).

### Central role of ser15 phosphorylation

Finally, p53 has been estimated to undergo about 60 different PTMs (53). More recently, however, an additional 150 PTMs, not previously reported, were identified on p53 in a single cell type by mass spectrometry (54). In spite of this extensive list of modifications, and the corresponding eye-watering number of permutations that may exist, Ser15, one of the first discovered sites of modification in p53, clearly plays a fundamentally important role in p53 function and biology—more so given that it is a primary target of certain stress stimuli (such as DNA damage) and can trigger many subsequent functionally important PTMs of the p53 protein ((55) and references therein).
