# Editor’s Note on ‘Critical role for p53-serine 15 phosphorylation in stimulating transactivation at p53-responsive promoters’

**DOI:** 10.1093/nar/gkag241

**Published:** 2026-03-14

This an editor’s note regarding: Jayne Loughery, Miranda Cox, Linda M. Smith, David W. Meek, Critical role for p53-serine 15 phosphorylation in stimulating transactivation at p53-responsive promoters, *Nucleic Acids Research*, Volume 42, Issue 12, 8 July 2014, Pages 7666–7680, https://doi.org/10.1093/nar/gku501

In September 2025, the Editors were alerted to concerns about potential duplication of the UV–Actin panel in “U2OS dose responses” and the Nutlin–Actin panel in “HCT116+/+ dose” blots shown in Figure 1. These concerns had also been raised on PubPeer: https://pubpeer.com/publications/CE47B23B398889A950E8873F1DD6DF.

Although the Editors have been unable to contact the authors, they carefully examined the data in question. The panels appear very similar when resized; however, the blots themselves are exceptionally clean, and there are no background artifacts that could be used for definitive comparison. Additional checks did not reveal any irregularities, apart from the fact that the bands have a similar shape.

The Editors found no evidence of image manipulation. However, in the absence of original data, the Editors advise readers to examine Figure 1 with care.

**Julian E. Sale** and **Barry L. Stoddard**

Senior Executive Editors

